# Identification, Characterization, and Production Optimization of 6-Methoxy-1H-Indole-2-Carboxylic Acid Antifungal Metabolite Produced by *Bacillus toyonensis* Isolate OQ071612

**DOI:** 10.3390/microorganisms11122835

**Published:** 2023-11-22

**Authors:** Sayed E. El-Sayed, Neveen A. Abdelaziz, Amer Al Ali, Mohammad Y. Alshahrani, Khaled M. Aboshanab, Ghadir S. El-Housseiny

**Affiliations:** 1 Department of Microbiology and Immunology, Faculty of Pharmacy, Ahram Canadian University, Giza 12566, Egypt; sayed.emad@acu.edu.eg (S.E.E.-S.); neveen.abdelaziz@acu.edu.eg (N.A.A.); 2 Department of Clinical Laboratory Sciences, Faculty of Applied Medical Sciences, University of Bisha, 255, Al Nakhil, Bisha 67714, Saudi Arabia; ameralali@ub.edu.sa; 3 Department of Clinical Laboratory Sciences, College of Applied Medical Sciences, King Khalid University, Abha 61413, Saudi Arabia; moyahya@kku.edu.sa; 4 Department of Microbiology and Immunology, Faculty of Pharmacy, Ain Shams University, Cairo 11566, Egypt; ghadir.elhossaieny@pharma.asu.edu.eg

**Keywords:** *Bacillus toyonensis*, response surface methodology (RSM), central composite design (CCD), indole carboxylic acid, antifungal

## Abstract

Fungal infections currently pose a real threat to human lives. In the current study, soil bacterial isolates were screened for the production of antifungal compounds to combat human fungal pathogens. Notably, the bacterial F1 isolate exhibited antimycotic action towards the *Candida albicans* ATCC 10231 and *Aspergillus niger* clinical isolates. By employing phenotypic and molecular techniques, we identified the F1 isolate as the *Bacillus toyonensis* isolate OQ071612. The purified extract showed stability within a pH range of 6–7 and at temperatures of up to 50 °C. It demonstrated potential antifungal activity in the presence of various surfactants, detergents, and enzymes. The purified extract was identified as 6-methoxy-1H-Indole-2-carboxylic acid using advanced spectroscopic techniques. To optimize the antifungal metabolite production, we utilized response surface methodology (RSM) with a face-centered central composite design, considering nutritional and environmental variables. The optimal conditions were as follows: starch (5 g/L), peptone (5 g/L), agitation rate of 150 rpm, pH 6, and 40 °C temperature. A confirmatory experiment validated the accuracy of the optimization process, resulting in an approximately 3.49-fold increase in production. This is the first documented report on the production and characterization of 6-methoxy-1H-Indole-2-carboxylic acid (MICA) antifungal metabolite from *Bacillus toyonensis.*

## 1. Introduction

A variety of secondary metabolites, including antibiotics, antifungals, and siderophores, are produced by microbes in soil ecosystems to facilitate communication, competition, and interaction with other species in the environment [1]. Few culturable microbial taxa provide the bulk of known antibiotics, and little is known about most soil bacteria’s capacity for biosynthesis [1]. The majority of recognized bacterial natural products, including currently used antibiotics, are derived from the microbial isolates of the Actinobacteria, Proteobacteria, and *Bacillus*, which represent microorganisms that frequently form a minority in soil microbial communities [1]. These natural sources avoid the contamination of water and the environment and minimize the risk to animal and human health [2]. Many secondary metabolites with novel antifungal and antibacterial activities from different fungal sources have also been characterized [2]. Secondary metabolites produced by microorganisms cause the effective inhibition of the germination of conidia in various pathogenic fungi [3]. Recent studies reported the inhibitory role of secondary metabolites, including the antagonistic activity against *S. sclerotiorum* and *Alternaria dauci* fungal pathogens [2]. Fungal illnesses have grown in medical, veterinary, and ecological importance over the last half-century. Globally, the present burden of mycotic illnesses in humans has reached several million cases [4,5]. A variety of fungi can result in diseases. However, *Candida* sp. is probably the most commonly observed [6]. The increased use of immunosuppressive medication, invasive surgery, and medical devices in modern medicine has caused a higher prevalence of human fungal infections. Another contributing factor is the frequent use of broad-spectrum antibiotics and antifungal medications. While these drugs are essential for combating bacterial and fungal infections, their extensive use can disrupt the natural balance of microbial communities in the body, thereby increasing the risks of diarrhea and other fatal infections [7]. Collectively, the aforementioned factors have contributed to the ongoing rise of invasive fungal infections, emphasizing the need for enhanced preventive measures, early detection, and targeted treatment strategies to address this global health challenge. The situation became more dangerous and challenging during and after the 2019 coronavirus disease (COVID-19) pandemic, where COVID-19 patients admitted to intensive care units (ICU) had the same risk factors for invasive fungal infections, including chronic respiratory diseases, corticosteroid medication, intubation/mechanical ventilation, cytokine storm, etc. [8]. Many articles indicated co-infection caused by bacteria and fungi in ICU COVID-19 patients, with *Aspergillus* infections being among them [9,10]. Mucormycosis, also known as “Black Fungus”, has also been linked to COVID-19 patients, making up approximately 70% of all mucormycosis cases reported [11]. Moreover, when the pre- and post-COVID-19 eras are compared, there is a higher number of candidemia-COVID-19 co-infections [12]. Furthermore, *Aspergillus fumigatus* has been identified as one of the most common sources of fungal infections in severely ill COVID-19 patients [13].

*Bacillus toyonensis* is a member of the *Bacillus cereus* group—it is a spore-forming, Gram-positive organism [14]. It has enormous economic significance; for example, *Bacillus toyonensis* BCT-7112 spores have been utilized as probiotic supplements in animal nutrition [15]. It was previously reported to exhibit plant growth promotion, biodegradation, and probiotic and biocontrol properties [16,17,18]. Its biocontrol properties are rarely reported; however, Wang et al. reported its toyoncin-producing ability. Toyoncin is a bacteriocin (a class of natural macromolecular protein) that is commonly produced by bacteria, e.g., *Bacillus* spp and *Lactobacillus* spp [19,20]. Toyoncin is an indole carboxylic acid derivative that displays antibacterial action against two major foodborne pathogens, *B. cereus* (through limiting the growth of its spores) and *Listeria monocytogenes* [20]. It also causes cell membrane damage [20]. Indole carboxylic acid derivatives have been previously reported for their antifungal activity [21,22] and have previously been documented to be produced by algae [23] and bacteria, such as *Micromonospora* sp. [24] and *Sandaracinus amylolyticus* [25].

To create an optimized product with superior attributes and quality, software-based optimization methods are used [26]. Response surface methodology (RSM) is a statistical tool that applies lower-order polynomial equations to develop, improve, and optimize a process with many factors that influence the response [27]. RSM reduces the overall number of possible combinations, saving time and materials during experimentation [28]. Hence, it was deemed necessary to use such techniques for optimizing the production of 6-methoxy-1H-Indole-2-carboxylic acid (MICA) for the first time from *Bacillus toyonensis* OQ071612.

RSM via the multifactorial design has been previously applied in our and resulted in the maximum production of many valuable microbial products, including antibiotics [29,30], antifungal products, [28], biosurfactants [31], and probiotics [32]. It has been previously established that statistical growth condition optimization improves the yield and the activities of various secondary metabolites. Therefore, this study aimed to identify, characterize, and structurally elucidate an antifungal metabolite produced by *Bacillus toyonensis* OQ071612, followed by the optimization of key physiological and environmental factors affecting production to reach the maximum productivity of the respective antifungal metabolite.

## 2. Materials and Methods

### 2.1. Collection and Phylogenetic Analysis of the F1 Strain

A soil isolate, coded F1, was recovered from a soil sample during a screening program previously conducted in our lab [33]. The strain was identified microscopically, biochemically, and via molecular techniques. The extraction of chromosomal DNA was performed in order to amplify and sequence the 16 S ribosomal RNA (16 S rRNA), as previously reported [34]. The final consensus sequence was obtained using the Staden package program version 2 (http://staden.sourceforge.net/) (accessed on 30 August 2023) and was analyzed using BLASTn (http://blast.ncbi.nlm.nih.gov/Blast.cgi (accessed on 30 August 2023). The MEGA X software (version 11) was used for the evolutionary and phylogenetic analyses [35]. The isolate was deposited in the Culture Collection Ain Shams University (CCASU) (http://ccinfo.wdcm.org/collection/by_id/1186) (accessed on 2 September 2023).under the accession code *Bacillus toyonensis* isolate CCASU-2023-OQ071612.

### 2.2. Evaluation of the Antifungal Activity

The isolate antifungal activity was demonstrated using the agar cross-streak technique against *Candida albicans* ATCC 10231, as was previously reported [36,37]. A dual culture technique was used for screening against *A. niger* [36,37]. After a 7-day preculture of *A. niger* on SDA (Sabouraud dextrose agar medium), mycelial fragments (0.5 × 0.5 cm) were placed on another plate on which the F1 isolate was co-cultivated [38]. The zone of inhibition and the percentage inhibition of fungal radial growth were calculated after incubation 7 days at 28 °C, as previously reported [39,40].

### 2.3. Evaluation of the Fungicidal Activity

To evaluate whether the antifungal activity was fungistatic or fungicidal against *A. niger*, agar plug was taken from the zones of inhibition area, added to a new SDA plate, followed by incubation at 28 °C for a week, and the growth of *A. niger* was monitored as previously described [40,41,42]. If the growth of *A. niger* resumed, this indicated that the metabolite had fungistatic properties, meaning it inhibited the growth of the fungus without killing it. *A. niger* was also cultured on a plain SDA plate to show its normal growth, and this served as a positive control.

### 2.4. Active Metabolite Production

A basal production medium was formulated, as previously stated by Singh et al. [39], consisting of various components such as glucose, Na_2_HPO_4_, K_2_HPO_4_, NH_4_Cl, NaCl, MgSO_4_, and CaCl_2_, at a pH 7 [43]. The isolate was inoculated into the medium at a count of 1 × 10^7^ CFU/mL, and the optimal incubation time was determined by preparing seven flasks of inoculated medium and sampling one flask daily for a week. Then, aliquots were centrifuged for 10 min at 51,610× *g* and the supernatant was tested for antifungal metabolite production against Candida albicans ATCC 10231 using the agar cup–plate technique. The resulting inhibition zones were measured each day and the largest value indicated optimum incubation time [44].

### 2.5. Testing the Intracellular and Extracellular Nature of the Antifungal Metabolites

To separate extracellular metabolites from the cell pellet, a micro-centrifuge (Centurion Scientific-K240R, West Sussex, UK) was used to centrifuge 1 mL of the culture for ten minutes at 51,610× *g*. The supernatant was subsequently tested on SDA plates. This was carried out on the day when the production of antifungal metabolites was at its peak, as determined by the zones of inhibition. The average diameter of the zones of inhibition was calculated from 3 replicates of each experiment. The plain production media was treated similarly to serve as a control plate [44].

In order to test the intracellular metabolite, the cells that were pelleted during centrifugation were washed by 1 mL sterile saline and resuspended in 200 μL of cell lysis buffer (Thermo Scientific™ B-PER™, Waltham, MA, USA) and vortexed (Benchmixer™ vortex mixer, Sayreville, NJ, USA). Afterwards, the pellets were incubated on ice for half an hour. Cells were then placed in a 1.5 mL Eppendorf tube and gently moved under the tip of the sonicator probe (Ultrasonic homogenizer, model 300 VT, 115 V/60 HZ (Anhui Zhongke Duling Commercial Appliance C, Hefei, China). The samples were sonicated at a frequency of 20 kHz for 30 s with 1 min stop intervals on ice between each run (pulsing mode). The procedure was repeated 4–5 times. Samples were then cooled on ice for 5 min and centrifuged for 15 min at 51,610× *g* to pellet debris. The supernatant (cell free lysate) was taken to a new tube and was tested, as mentioned above [45]. Plain production media was again treated similarly and served as the control [45]. The results were compared to those of the extracellular activity.

### 2.6. Extraction and Purification

After the centrifugation of the culture broth (20,160× *g* for 30 min), the culture supernatant was filtered and stored at 4 °C for additional analysis. To investigate the peptide nature of the metabolite, ammonium sulphate was mixed with the supernatant, incubated at 4 °C with shaking overnight, and was monitored for any peptide precipitation [43].

The extraction of the antifungal metabolites was carried out as previously reported [46,47]. Briefly, equal amounts of various solvents, including diethyl ether, methanol, ethanol, chloroform, hexane, ethyl acetate (EA), acetone, dichloromethane, and n-butanol (PioChem, Giza, Egypt; all of HPLC grade) were used at a ratio 1:1 (*v*/*v*) to extract the metabolite(s) [48]. After shaking vigorously for ten minutes at room temperature, each mixture was poured into a separating funnel and left to stand until the organic and aqueous phases were distinct, then the organic phase was collected in separate containers. This process was repeated thrice, and the organic phase later evaporated to dryness under reduced pressure using a rotary evaporator at 45 °C (Staurt RE300; Kaison Co., Essex, UK) to yield different organic solvents extracts [49]. The antifungal activity of the extracts was tested using the agar well diffusion technique, and negative controls were created using the corresponding solvents [48,50]. The solvent that showed the maximum extraction of the antifungal metabolite—expressed by the largest zone of inhibition—was chosen for subsequent procedures [51].

The purification of the antifungal metabolite was carried out using chromatography silica gel column (3.5 × 80 cm), mesh size 60–120 (Merck, Darmstadt, Germany). The linear gradients of increasing polarity for the chloroform and ethyl acetate solvents were used, and the flow rate was adjusted to 1 mL/min [52]. Fractions were collected at regular intervals, spotted on thin layer chromatography (TLC) plates and developed using the same solvent system. Elutions with similar retardation factors (RF) were pooled together and pooled fractions (PFs) were dried, weighed, and checked for their antifungal activity using the bioautography method against *C. albicans* ATCC 10231 [53]. The fractions with the greatest antifungal activity were purified once more using the column chromatography method, and the purities were checked using TLC plates. Purity was confirmed via the visualization of a single spot under a UV lamp (UVitec^®,^ Novolab, Geraardsbergen, Belgium) at 254 nm and 365 nm. This made it possible to prove the antifungal compound’s purity in the fractions, which was crucial to determine how effective they would be as therapeutic agents [54].

### 2.7. Physicochemical Properties of the Antifungal Metabolite(s)

In order to ascertain the antifungal metabolite’s thermal durability, the following temperatures were applied to 100 µL of the purified antifungal extract (100 µL g/mL) in 7 screw-capped ampoules for one hour: 30 °C, 40 °C, 50 °C, 60 °C, 70 °C, and 80 °C. The final ampoule was autoclaved for 15 min at 121 °C [55]. Additionally, 1 mL of 0.1 M phosphate buffer at various pH levels (5.7–8.0) was added to 1 mL of the tested metabolite for an hour to assess the impact of pH on the stability of the metabolite. The effects of various detergents, including sodium dodecyl sulphate (SDS), Tween 20, Tween 40, and Tween 80, were also studied. A total of 10 ml of aqueous detergent solution (10 mg/mL) was combined with 100 µL of the antifungal solution and incubated at 30 °C for six hours [56]. The effect of enzymes was examined by combining 100 µL of the antifungal solution with 10 mL of the enzyme solutions (1 mg/mL), including proteinase K, alpha amylase, and lysozyme (all from Sigma-Aldrich) and were incubated at 30 °C for 3 h. As controls, tubes containing the antifungal solution without any enzymes or detergents were used. Moreover, control tubes containing the enzymes and detergents only without the antifungal metabolite were also tested for any activity [56]. Following the aforementioned treatments, the metabolite’s residual antifungal activities were examined using cup–plate method (100 µL/cup), and the average values of the inhibitory zones were recorded [55].

### 2.8. Spectroscopic Analysis

The spectroscopic analysis was carried out at the Drug Discovery and Development Research Centre (DDDC), Ain Shams University Cairo, Egypt. A total of 1 mg of the sample was dissolved in 10 mL of DMSO and a Shimadzu UV-1800 spectrophotometer was used to record the ultraviolet (UV) spectrum in a range of 200–400 nm. (Two-dimensional) NMR spectroscopic data were measured at room temperature in methanol on a Bruker^®^ Avance III HD 400 MHz spectrometer, Bremen, Germany, equipped with a 5 mm broad-band multinuclear (PABBO) probe. The chemical shifts were reported in parts per million (ppm) relative to TMS (δ = 0.0), used as internal standard, and the coupling constants (J) were reported in Hertz (Hz). All the ^1^ H and ^13^C signals were assigned using the ^1^H—^1^H COSY, ^1^H—^13^C HSQC, and ^1^H—^13^C HMBC experiments. The electrospray ionization mass spectrometry (ESI–MS) spectrum analysis of the isolated compound was recorded on a Shimadzu LC-MS 8045 (Shimadzu, Japan).

### 2.9. Factors Affecting Antifungal Metabolite Production

The ‘one-factor-at-a-time’ (OFAT) method was employed for the primary screening of important factors affecting production. These included various carbon and nitrogen sources. Glucose already present in the medium was replaced with 4 other carbon sources (glycerol, starch, lactose, or sucrose), all at 5 g/L. Similarly, the ammonium chloride (nitrogen source) was replaced with 4 other nitrogen sources (casein, urea, yeast, or peptone) all at 1 g/L, using the best carbon source chosen. Each experiment was carried out in triplicate, and the matching inhibition zones’ mean values were calculated [43]. The optimal nitrogen and carbon sources were chosen for the next assays.

### 2.10. Production Optimization Using RSM

For the optimization procedure, a face-centered central composite design (CCD) was used [57]. The factors A, B, C, D, and E were the carbon source, nitrogen source, temperature, pH, and agitation rate, respectively. As shown in Table 1, three levels of each factor were evaluated. The inhibition zone diameter (response value) was determined after 4 days. Utilizing Design Expert^®^ v. 11.0 (Design Expert^®^ Software, Stat-Ease Inc., Statistics Made Easy, Minneapolis, MN, USA), the experiments were designed. A total of 22 runs were completed. To clarify the correlations between the five experimental variables and the inhibition zone widths, three-dimensional response surface graphs were plotted.

### 2.11. Confirmation of the Model Used for Optimization

The recommended optimal conditions were obtained from the Design Expert^®^ Software, and a further experiment was run using these optimum parameters. The concentration of antifungal metabolites acquired under these settings was compared to that obtained under the basal conditions.

### 2.12. Statistical Analysis

Every experiment was performed in triplicate, and the results shown were the mean with the standard error. Experimental design and graphical plots were created using Design Expert^®^ v. 11.0. ANOVA analysis was employed to statistically validate the experimental data.

## 3. Results

### 3.1. Antifungal Activity and the Identification of the F1 Isolate

The F1 isolate showed significant antifungal activity against *C. albicans* (IZ = 12.6 ± 0.58 mm) (Figure S1) and radial mycelial growth inhibition of 61.3% using the dual culture method against *A. niger* (Figure S2). The F1 isolate was found to be a Gram-positive, spore-forming bacteria with the following biochemical reactions: positive for citrate utilization, urease synthesis, and starch and casein hydrolysis; negative for catalase and gelatin liquefaction.

The 16 S ribosomal RNA sequence of F1 isolate was obtained and deposited in GenBank (NCBI code, OQ071612 (https://www.ncbi.nlm.nih.gov/nuccore/OQ071612 (accessed on 30 August 2023). A BLASTn similarity search with *Bacillus toyonensis* OQ071612 as the query sequence found a 98.8% similarity with the *Bacillus toyonensis* strain G (NR _025357.1). Their close kinship was further supported by the phylogenetic tree, which was created (Figure S3). Accordingly, the F1 isolate was identified as *Bacillus toyonensis* OQ071612.

### 3.2. Characterization of the Antifungal Metabolite

It was observed that no growth of *A. niger* could be observed upon culturing the agar plug that was excised from the edge of the inhibition zone, confirming that the antifungal metabolite had a fungicidal activity against *A. niger*. It was shown that an incubation period of 4 days was the optimum time for the highest production of the respective antifungal metabolite (Figure S4). Production on day 4 was significantly higher (*p* < 0.05) than production on all other days, except day 3. The antifungal metabolite was found to be secreted in the culture broth of *Bacillus toyonensis* OQ071612 in larger amounts than those obtained intracellularly, as indicated by the resulting mean inhibition zones (Figure S5). This proved that the antifungal metabolite is excreted extracellularly in a reasonable quantity.

### 3.3. Solvent Extraction

Ammonium sulphate did not precipitate the creation of any peptides from the culture supernatant. As displayed in Table 2, ethyl acetate was the optimum solvent producing a dry weight of 0.28 mg from one ml culture broth and inhibition zones of 14.4 and 14.1 mm against the *C. albicans* ATCC10231 and *A. niger* clinical isolates, respectively. Based on their TLC characteristics, 145 fractions were gathered and aggregated into 22 pooled fractions (PFs). The bioautography assay revealed the highest antifungal activity for PFs 17, 18, and 19. Other PFs displayed little or no activity (Table 3).

The elutes 121–126 forming PF 18 were obtained using a chloroform/ethyl acetate solvent system with a ratio of 15:85 and exhibited the strongest antifungal activity, as shown in the bioautography assay (Figure S6). Figure S7 confirms the purity through the visualization of a single spot on TLC plate under a UV lamp (UVitec^®^) at 254 nm and 365 nm. Figures S8 and S9 show the HPLC chromatogram and the positive ESI–MS spectrum analysis of the isolated compound, respectively. Figure S10 shows the linear relationship between the various concentrations and the inhibition zones. The following equation was a representative of this linear relationship: y = 0.1109x + 9.1094 (Y = inhibition zone diameter in mm, X = concentration of the best active fraction in μg/mL).

### 3.4. Physicochemical Properties of the Antifungal Metabolite(s)

**Figure 1:**
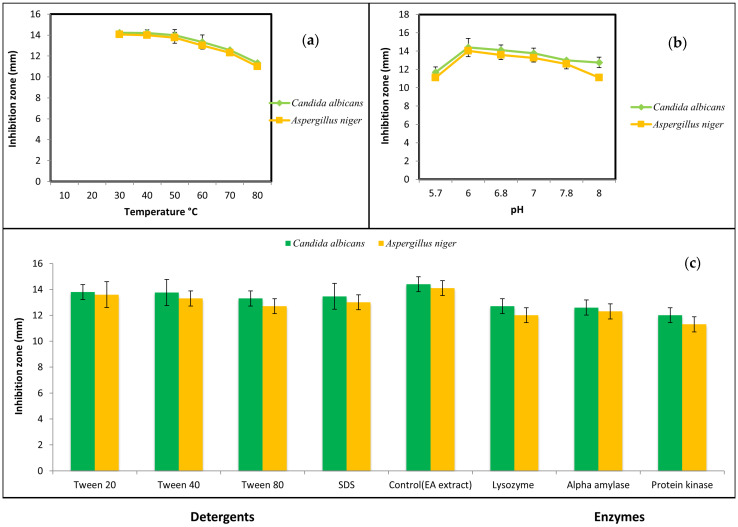
Stability of the pure the antifungal metabolite under various (**a**) temperatures, (**b**) pH, and (**c**) detergents and enzymes.

The bioactive metabolite of *B. toyonensis* preserved its activity up to 50 °C. Higher temperatures resulted in a declined activity while autoclaving at 121 °C for 15 min caused a complete loss in activity (Figure 1a). Regarding pH, the metabolite preserved its action over a pH range from 6–7 (Figure 1b). Likewise, activity was not influenced by Tween 20, 40, and 80; SDS; alpha amylase; proteinase K; or lysozyme (Figure 1c). As controls, these enzymes and detergents showed no activity against the tested fungus.

### 3.5. Spectroscopic Analysis

**Figure 2:**
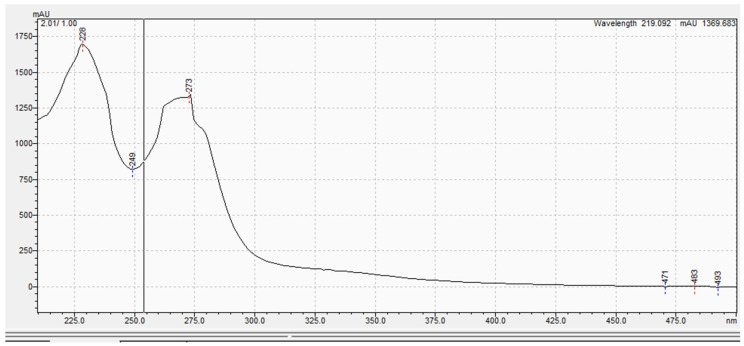
The ultraviolet (UV) absorption spectrum of the purified compound.

**Figure 3:**
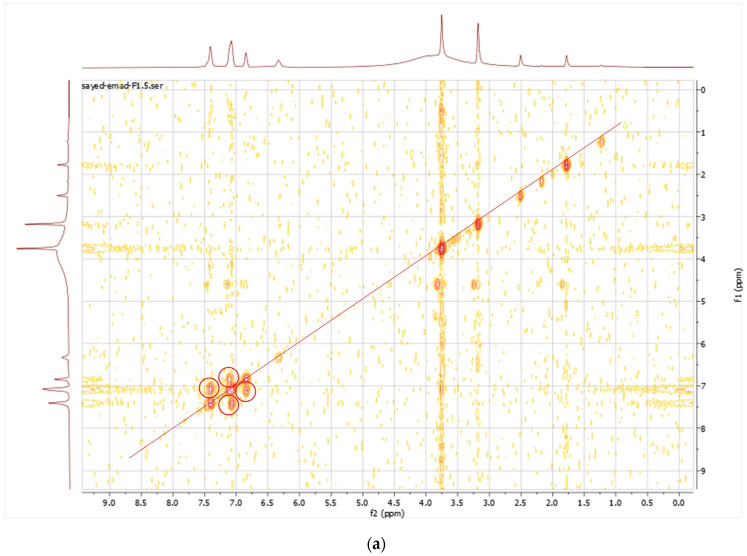

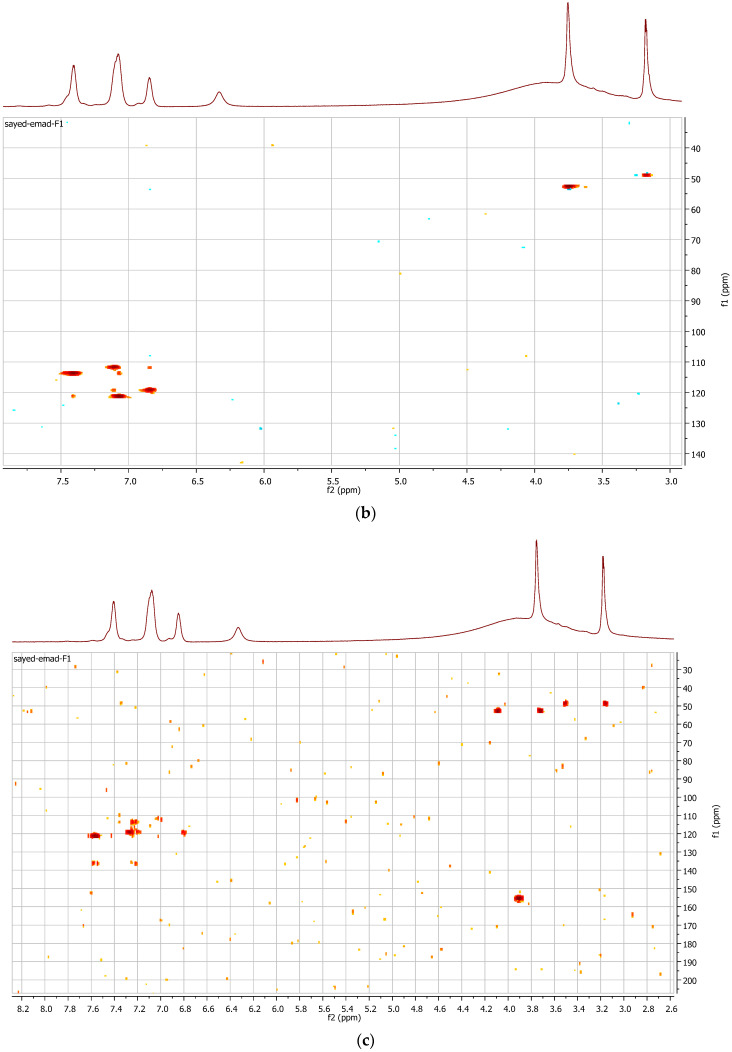
(**a**) COSY spectrum of the purified compound; (**b**) HSQC spectrum of the purified compound; (**c**) HMBC spectrum of the purified compound.

The purified metabolite appeared as an off-white amorphous solid powder. The ultraviolet (UV) absorption spectrum documented a λmax at 273 nm (Figure 2). For further confirmation of the respective λmax, Figure S11 demonstrates different dilutions (2–18 µg/mL) prepared for the respective compounds, and their recorded UV absorbances at the chosen lambda maximum HPLC-ESIMS spectrum of the purified metabolite revealed a pseudomolecular ion peak at *m*/*z* 192 (M+H) (Figures S8 and S9). The ^1^H NMR spectrum, COSY (Figure 3a), displayed correlations between coupled hydrogens, while HSQC (Figure 3b) allowed for the assignment of carbons with connected protons. Connectivity between all groups is listed in Table 4 along with HMBC assignments for long range ^1^H—^13^C couplings (Figure 3c).

**Figure 4:**
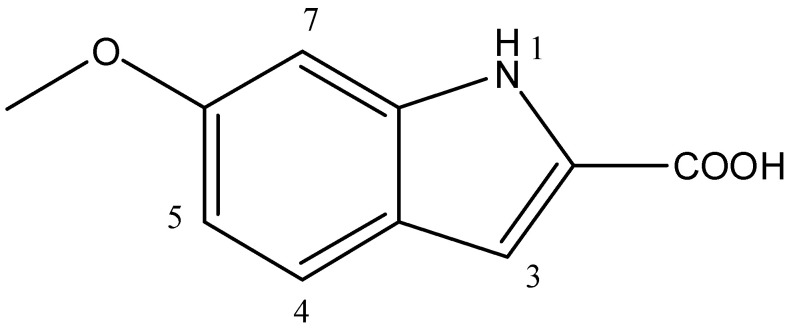
Chemical structure of 6-methoxy-1H-Indole-2-carboxylic acid produced by *Bacillus toyonensis* OQ071612.

Based on the results obtained from the advanced spectroscopic analysis, the structure of the metabolite was established as 6-methoxy-1H-Indole-2-carboxylic acid (Figure 4).

### 3.6. Effect of the Composition of the Culture Media

**Figure 5:**
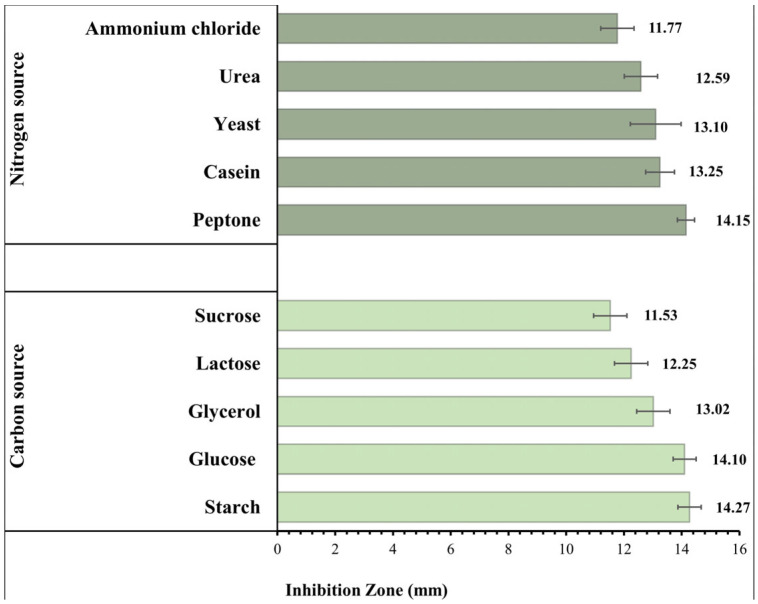
Effect of different carbon and nitrogen sources on the antifungal activity of *Bacillus toyonensis* OQ071612 against *C. albicans* ATCC 10231.

The impact of various C and N sources on the IZ diameters produced by *B. toyonensis* against *C. albicans* is shown in Figure 5. Large inhibitory zones showed an increase in the *B. toyonensis* synthesis of the antifungal metabolite when compared to that obtained in normal production media (IZ diameter = 12.56 mm). Starch (5 g/L) and peptone (1 g/L) were chosen for further research since they influenced the maximum production of the active metabolite.

### 3.7. Optimization of the Bacterial Culture Conditions Using RSM

The antifungal metabolite production was well optimized, and the values of the assessed factors are displayed in Table 5. The resulting equation is as follows:

$$1/\sqrt{IZ}=0.313021-0.014527\times \mathrm{starch}-0.004163\times \mathrm{peptone}-0.000532\mathrm{temperature}+0.002966\times \mathrm{pH}+0.000019\times \mathrm{agitation}\;\mathrm{rate}.$$

The significance of the generated model was assessed using ANOVA (Table 6). The model’s F-value was 183.58 (*p*-value < 0.0001), demonstrating its significance. The significant model terms were A, B, C, and D, which all have *p*-values under 0.05. Additionally, a low coefficient of variation of 1.36 was found, indicating the strong dependability of the experimental values. The coefficient of determination, R^2^, which was 0.9829, indicates that the model could account for 98.2% of the response variability. Additionally, a respectable adj R^2^ of 0.9775, closely agreeing with the pred R^2^ of 0.9712, was attained. Finally, the adequate precision, or signal to noise ratio, was equal to 44.804. This model was therefore appropriate for navigating the design space.

**Figure 6:**
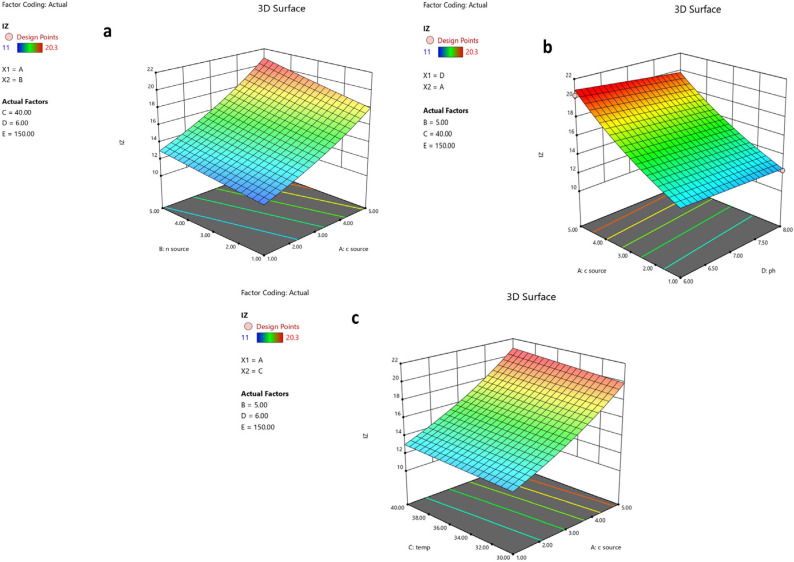
The 3D response surfaces of the five parameters affecting activity of metabolite produced by *B. toyonensis* OQ071612. When two factors where plotted, the other three factors were kept at optimum levels. (**a**) The effect of the carbon source and nitrogen source, (**b**) the effect of the pH and carbon source, and (**c**) the effect of the carbon source and temperature on antifungal metabolite production.

The 3D plots in Figure 6a–c illustrate how the IZ is influenced by any two factors. The suggested ideal conditions for maximum production of the 6-methoxy-1H-Indole-2-carboxylic acid were determined to be starch 5 g/L, peptone 5 g/L, an agitation rate of 150 rpm, a pH of 6, and a temperature of 40 °C.

**Figure 7:**
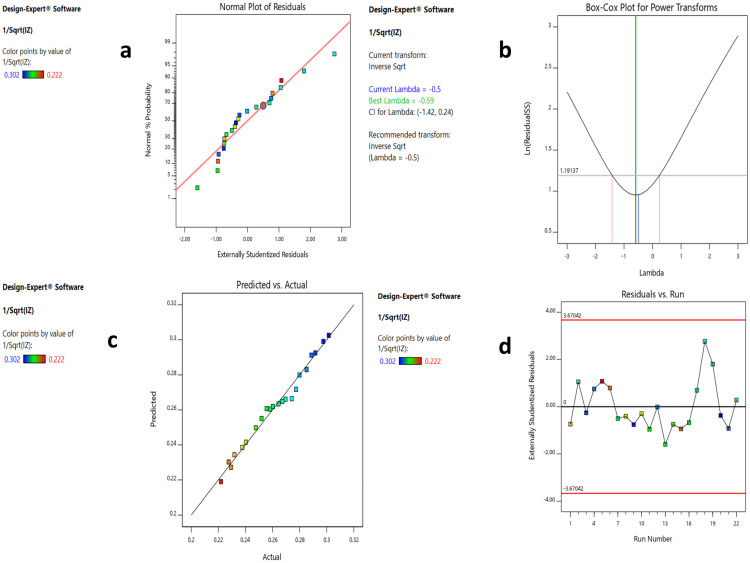
Results of the four model diagnostic plots of the CCD model. (**a**–**d**) explanations. (**a**) Normal plot of residuals showing linear pattern. (**b**) Box-Cox plot showing a recommended transformation of lambda to inverse square root. (**c**) Predicted vs. actual plot showing points close to the straight line. (**d**) Residual vs. run plot showing a random scatter around zero.

The probability of residuals, the Box–Cox transformation and the predicted versus actual values plots are displayed in Figure 7a–c, suggesting the linear pattern, the necessary transformation, and an acceptable correlation between the predicted and the actual data, respectively. In Figure 7d, the residual vs. run plot shows that the points were randomly distributed around zero, which validates the generated model.

### 3.8. Confirmatory Experiment Using Optimal Conditions

**Figure 8:**
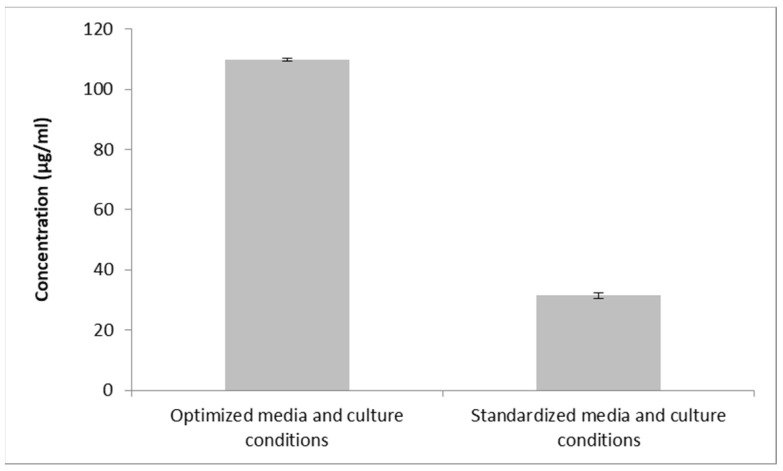
Antifungal metabolite concentration using optimized conditions, as compared the unoptimized conditions.

The confirmation trial was conducted using the recommended optimal levels, yielding a maximal inhibition zone of 21.3 mm or a concentration of 109.92 µg/mL. As a result, the concentration of the metabolite was 3.49 times higher under the optimum conditions than it was under the unoptimized ones (12.6 mm, corresponding to 31.48 µg/mL) (Figure 8).

## 4. Discussion

The antifungal properties of the soil F1 isolate were examined in the current investigation. Using the cross-streak and agar well diffusion procedures, significant antifungal activity against *C. albicans* and *A. niger* were established. The F1 isolate was identified as *B. toyonensis* based on its morphology, biochemical characteristics, and 16 S ribosomal RNA sequencing (NCBI GenBank accession number OQ071612). This Gram-positive isolate belonged to *Bacillus*, which is frequently cited as a source of beneficial bioactive compounds. The genus *Bacillus* are known as a bio-factor in antifungal secondary metabolites that combat many pathogenic fungal strains [58]. Previously, a strain of *B. toyonensis* obtained from the spontaneous cocoa beans fermentation showed a significant fungistatic activity against *Aspergillus carbonarius* [59]. In another study, *B. toyonensis* exhibited antifungal activities against many plant fungal pathogens [60]. Moreover, it showed many antimicrobial properties [61] and targeted activity against *B. cereus* and *Listeria monocytogenes* through its toyoncin production ability [20]. A soil-recovered strain of *B. toyonensis* has also shown promising insecticidal activities in former studies [62].

In the current study, the respective metabolite was found to be formed extracellularly, and ethyl acetate was used to extract it with a considerably higher yield (0.28 mg/mL). A silica gel thin layer chromatogram was used to identify the active fraction (PF18) with the highest antifungal activity, and bioautography testing against *C. albicans* served as confirmation. The metabolite’s primary utility in pharmacotherapy is that it demonstrated fungicidal activity [33]. We identified this compound as 6-methoxy-1H-Indole-2-carboxylic acid using 1D- and 2D-NMR and LC-PDA-MS spectral studies. This compound contains the indole carboxylic acid moiety, which had been previously reported for its antifungal and antimicrobial activities [63] from various microorganisms and algae [23]. Furthermore, 4-[(1E)-3-methylbuta-1,3-dienyl]-1H-indole-3-carbaldehyde (indiacene A) and -[(1E,3E)-4-chloro-3-methylbuta-1,3-dienyl]-1H-indole-3-carbaldehyde (indiacene B)-prenyl indoles harboring the same moiety were extracted and purified from the culture broth of the Myxobacterium *Sandaracinus amylolyticus* [25]. Moreover, 3-methyl-1Hindole- 2-carboxylic acid and 1H-indole-3-carboxaldehyde were previously isolated from the culture broth of *Micromonospora* sp. and showed antagonistic activity against many pathogens, including *Staphylococcus aureus*, *Enterococcus faecium*, and *Escherichia coli* [64].

Even though 1H-Indole-2-carboxylic acid derivatives have been reported in the literature, this is the first report denoting its production by *B. toyonensis* OQ071612. The method through which secondary metabolites are produced by microorganisms is complex [65]. The quality and quantity of the produced metabolites can be affected by slight modifications to the medium components and/or circumstances. Hence, the best sources of carbon and nitrogen were first identified using the OFAT method. Carbon and nitrogen are essential components needed for the growth of bacteria and the synthesis of metabolites [64]. In bacterial fermentation operations, carbohydrates are typically employed as carbon sources, but nitrogen sources can be either organic or inorganic [64,66]. In this investigation, the best media components for maximizing metabolite production were identified to be starch and peptone. Starch is agreed to be an important medium component for antifungal compounds production from microorganisms. Maximum growth and antibiotic production was found to occur when starch was used as the only C source [67]. This is in accordance with Shakeel et al. (2016), who reported that starch was the optimum carbon source for the *Streptomyces platensis* 3–10 growth [68]. Jacob et al. (2014) reported that antibacterial metabolites were produced at a higher level with starch as the carbon source in *Streptomyces nogalater* NIIST A30 [69]. Chen et al. (2022) showed that the antifungal metabolites production was significantly improved with the addition of soluble starch in *S. alfalfae* [70]. Carbon sources are essential components not only in constructing cellular materials but also when utilized as energy sources [71]. Nitrogen, on the other hand, is crucial for nucleic acid and protein production, which are the raw materials that create cellular metabolites [72]. In fermentation, the interaction between cell growth and secondary metabolite secretion is critically affected by the growth-limiting nutrients at certain concentrations [68]. Hence, selection is an essential step in the large-scale production of bacterial secondary antifungal metabolites [70]. Peptone is a complex mixture of peptides, with a small content of free amino acids, obtained from the enzymatic hydrolysis of animal proteins. The peptides play an principal role in cell metabolism by supplying essential amino acids and metabolic energy, and this explains the significant effect of peptone concentration on the metabolite production [73]. Chattopadhyay et al. (1997) proposed peptone as a nitrogen source for the maximum antifungal activity of *Streptomyces rochei* G 164 [74]. However, for different strains of the same species, e.g., ACTA1551, Kanini et al. (2013) found soy peptone to be the most appropriate source of nitrogen for antifungal activity against *Fusarium oxysporum* f. sp. *Lycopersici* [75]. Moreover, peptone was considered an appropriate medium component for antifungal production from *Streptomyces chilikensis* ACITM-1 [67].

To improve and optimize the production of the antifungal metabolite, we investigated the growth condition variables and their interactions using a powerful statistical method: RSM. RSM has frequently been employed to enhance the production of antifungal metabolites by several soil bacterial isolates [76]. This method creates interactive plots and model equations that show how various parameters affect a certain reaction [77]. This is all accomplished with reasonable resources and time, as opposed to the OFAT approach. The CCD, one of the most widely used and effective designs, is perfect for subsequent analysis, with a manageable number of runs [78]. To investigate the effects of five variables (starch, peptone, pH, temperature, and agitation) on production, a sum of 22 runs were performed. The significance of the model was assessed using ANOVA, which offers an indication of the sources of variance [79]. The significant nature of the model (*p* value < 0.0001) was demonstrated using a large F-value (Fisher’s value = 183.58), which compares the mean square values of the model and residual errors [61]. The determination coefficient, R^2^, adjusted R^2^, and predicted R^2^ values, all being near 1, generally indicate excellent correlation for any regression model [80,81]. The R^2^ value (0.982) proved a good fit between observed and predicted responses. The obtained adj R^2^ (0.9775), which determines whether extra input factors contribute to the model, was also acceptable. The ability of the model to precisely predict a response, or pred R^2^ (0.9712), should vary from the adj R2 by a value of no more than 0.2, which was the case here [75]. The signal-to-noise ratio, or adequate precision, was 44.804, which is greater than 4 and suggests a good signal. Furthermore, the reliability of the trials was indicated by the low coefficient of variation (CV%) of 1.36 [82]. The *p*-value was used to identify the factors that had a significant impact on the metabolite. Starch (A), peptone (B), temperature (C), and pH (D) were all significant (*p* < 0.05), as presented in the results, showing that these four variables influence the production. The model was displayed as three-dimensional plots, a three-dimensional depiction of the response for chosen components, to help understand the influence of the factors on the antifungal metabolite yield. These charts show how two variables interact, which makes it possible to determine the optimum experimental setup directly. As shown in the color code, a red zone indicates maximum inhibition zones, and thus, maximum production, while a blue zone indicates the minimum production of the antifungal metabolite. These 3D plots, together with the numerical optimization function in the software, determine the optimum conditions for maximum antifungal metabolite production, and these conditions were verified experimentally. Our design and optimization model were validated because the actual experimental results were in good agreement with the projected outcomes from the software for this experiment. The metabolite produced a maximal inhibition zone of 21.3 mm, which is equal to 109.92 µg/mL. Therefore, compared to unoptimized conditions (31.475 µg /mL), optimization in our investigation was able to increase the *B. toyonensis* OQ071612 production of the metabolite by approximately 3.49-fold.

Graphical model diagnostics are plotted by the software (Design Expert^®^ v. 11.0, Minneapolis, MN, USA) to verify ANOVA results. These plots include normal plots of residuals (Figure 7a), which reveal whether the residuals (difference between an actual and predicted value) follow a normal distribution, i.e., a straight line or not. The Box–Cox plot recommends the correct power transformation, based on the best lambda value obtained at the lowest point of the curve. In our case, the software recommended a transformation to the inverse square root, as shown in Figure 7b. Another diagnostic plot is the predicted vs. actual plot, which displays the values not easily predicted by the model (Figure 7c). Finally, the residual vs. run plot (Figure 7d) identifies trends that indicate lurking variables that influence the response and ruin the analysis.

## 5. Conclusions

This study demonstrates the necessity of the continuous deciphering of promising antifungal metabolites from natural sources. Herein, a soil isolate exhibited promising antifungal activities against *Candida albicans* and *A. niger* was identified as *Bacillus toyonensis* OQ071612. The produced antifungal metabolite exhibited promising fungicidal properties and stability within a pH range of 6–7, temperatures of up to 50 °C, and in the presence of various surfactants, detergents, and enzymes. The antifungal molecule was chemically identified as 6-methoxy-1H-Indole-2-carboxylic acid, using advanced spectroscopic techniques. Its production was statistically optimized using response surface methodology (RSM) with a central composite design, resulting in an approximately 3.49-fold increase. This is the first report on the production optimization of the respective chemical moiety from *Bacillus toyonensis* against human fungal pathogens.

## Figures and Tables

**Table 1:** Parameters used for the face-centered central composite design.

Factor	Symbol	Level		
		−1	0	+1
Starch (g/L)	A	1	3	5
Peptone (g/L)	B	1	3	5
Temperature (°C)	C	30	35	40
pH	D	6	7	8
Agitation rate (rpm)	E	150	225	300

**Table 2:** Extraction yield and mean inhibition zones of different solvents.

Solvent	Extraction Yield (mg/mL)	Mean Inhibition Zones (mm) ± SD	
		*C. albicans*	*A. niger*
Ethyl acetate	0.28	14.4 ± 0.32	14.1 ± 0.58
Chloroform	0.26	14.11 ± 0.16	14.0 ± 0
n-hexane Dichloromethane	0.22 0.19	14.0 ± 0 13.2 ± 0.58	13.9 ± 0.72 13.0 ± 0
Acetone	0.15	12.43 ± 0.52	12.1 ± 0.15
Diethyl ether	0.11	11.3 ± 0.42	11.4 ± 0.33
n-butanol(n-butyl alcohol)	-	-	-
Ethanol	-	-	-
Methanol	-	-	-

**Table 3:** Solvent ratio for each pooled fraction, numbers of recovered elutes, and corresponding zones of inhibition.

Pooled Fractions (PFs)	Ratio of Chloroform: Ethyl Acetate	Elutes Recovered	Retardation Factor (RF)	Dry Weight of Each PF (mg)	Mean Inhibition Zones (mm) ± SD	
					*C. albicans*	*A. niger*
1	Chloroform 100%	1–4	0.87	0.63	-	-
2	95:5	5–12	0.32	0.25	-	-
3	90:10	13–19	0.44	0.71	11.0 ± 0	-
4	85:15	20–27	0.57	0.34	11.3 ± 0.14	11.0 ± 0
5	80:20	28–44	0.23	0.65	11.22 ± 0.28	11.3 ± 0.50
6	75:25	45–54	0.85	0.11	12.33 ± 0.61	12.11 ± 0.52
7	70:30	55–62	0.79	0.42	12.5 ± 0.12	12.26 ± 0.64
8	65:35	63–72	0.65	0.22	12.58 ± 0.21	11.6 ± 0.0.58
9	60:40	73–75	0.52	0.64	13.34 ± 0.64	13.12 ± 0.18
10	55:45	76–83	0.89	0.89	13.76 ± 0.30	12.3 ± 0.43
11	50:50	84–86	0.18	0.90	13.6 ± 0.48	13.1 ± 0.11
12	45:55	87–90	0.38	0.40	13.3 ± 0.23	12.76 ± 0.68
13	40:60	91–96	0.76	0.56	13.76 ± 0.12	13.4 ± 0.71
14	35:65	97–101	0.41	0.71	14.3 ± 0.64	14.0 ± 0
15	30:70	102–108	0.58	0.65	14.7 ± 0.56	14.3 ± 0.33
16	25:75	109–114	0.62	0.79	14.7 ± 0.16	14.6 ± 0.18
17	20:80	115–120	0.11	0.40	15.6 ± 0.44	15.4 ± 0.35
18	15:85	121–126	0.21	0.81	15.8 ± 0.72	15.6 ± 0.62
19	10:90	127–134	0.25	0.21	15.4 ± 0.14	15.1 ± 0.27
20	5:95	135–139	0.97	0.23	11.3 ± 0.33	11.1 ± 0.12
21	2.5:97.5	140–142	0.70	0.49	12.76 ± 0.54	12.4 ± 0.15
22	Ethylacetate	143–145	0.94	0.36	13.6 ± 0.32	13.3 ± 0.26

**Table 4:** ^1^H NMR and ^13^C NMR data of the pure antifungal metabolite.

Position	δ_H_ (MeOD, 400 MHz, J in Hz)	δ_C_ Obtained from, COSY, HMBC and HSQC Spectra (MeOD, 100 MHz)
1	(NH) 6.3 (s)	-
2	-	124.1 (C)
3	7.10 (s)	111.75 (CH)
4	7.41 (m)	114 (CH)
5	6.85 (m)	119.6 (CH)
6		156 (C)
7	7.08 (brs)	121.6
6-OCH3	3.76 (s)	53.5 (C)

Assignments were carried out based on COSY, HSQC, and HMBC experiments.

**Table 5:** Observed and predicted responses of the central composite design runs.

Run	A: Starch	B: Peptone	C: Temperature	D: pH	E: Agitation Rate	Observed Inhibition Zone (mm)	Predicted Inhibition Zone (mm)
1	5	3	35	7	225	18.6	18.09
2	3	3	30	7	225	13.76	14.46
3	1	3	35	7	225	11.76	11.43
4	1	5	40	8	150	12.3	12.59
5	5	5	40	6	150	20.3	20.01
6	5	5	30	6	300	19	18.94
7	3	3	35	7	150	14.76	14.99
8	5	1	40	8	150	17.3	17.25
9	1	1	30	6	150	11.3	10.73
10	5	1	40	6	300	17.7	17.54
11	3	5	35	7	225	15.76	15.77
12	1	5	40	6	300	12.76	12.89
13	3	3	40	7	225	15.3	15.07
14	5	1	30	8	300	16.3	16.17
15	5	5	30	8	150	19.3	18.65
16	3	3	35	6	225	15	15.14
17	3	3	35	7	300	14	14.53
18	3	3	35	8	225	13.3	14.38
19	3	1	35	7	225	13	13.76
20	1	1	40	8	300	11	10.12
21	1	5	30	8	300	12	11.52
22	3	3	35	7	225	14.3	14.76

**Table 6:** The results of ANOVA analysis of the CCD model.

Source	Sum of Squares	df	Mean Square	F-Value	*p*-Value	
Model	0.0117	5	0.0023	183.58	<0.0001	significant
A-Starch source	0.0106	1	0.0106	827.52	<0.0001	
B-peptone source	0.0009	1	0.0009	67.96	<0.0001	
C-Temperature	0.0001	1	0.0001	6.93	0.0181	
D-pH	0.0001	1	0.0001	8.62	0.0097	
E-Agitation rate	0.0000	1	0.0000	2.05	0.1718	
Residual	0.0002	16	0.0000			
Corrected Total	0.0119	21				

## Data Availability

Data are contained within the article and Supplementary File. The 16 S ribosomal DNA was deposited in the NCBI GenBank database under the accession code OQ071612.1; https://www.ncbi.nlm.nih.gov/nuccore/OQ071612 (accessed on 30 August 2023).

## Supplementary Materials

The following supporting information can be downloaded at: https://www.mdpi.com/article/10.3390/microorganisms11122835/s1, Figure S1: Cross-streak method of isolate (F1) against *Candida albicans* ATCC10231; Figure S2: Dual culture technique showing inhibitory effect of the isolate (F1) against *A. niger.* (a) shows clear zone between the edges of fungal mycelia and bacterial colonies. (b) shows the control, inoculated with *A. niger* without streaked bacteria. (c) is the control, showing the diffuse growth of *A.niger* in the presence of a non-productive bacteria; Figure S3: Molecular phylogenetic analysis of the query isolate *Bacillus toyonensis* OQ071612 strain (F1) using the maximum likelihood method based on the Tamura–Nei model in MEGA X; Figure S4: Effect of incubation time on the yield of the antifungal metabolite produced by *Bacillus toyonensis* OQ071612 against *Candida albicans* ATCC10231; Figure S5: Average inhibition zones of the tested intracellular and extracellular antifungal metabolite(s) produced by *Bacillus toyonensis* OQ071612 against *Candida albicans* ATCC10231; Figure S6: The bioautography method was used to determine the antifungal activity of the pooled fractions obtained from column chromatography fractionation against *C. albicans*. Plate (a) highlights the zone of inhibition caused by the fractions of the *B. toyonensis* metabolite with the most potent antifungal activity. Plate (b) shows the control plate; Figure S7: Visualization of the most active pooled fractions of the antifungal metabolite of *B. toyonensis* isolate OQ071612 under a UV lamp (UVitec^®^) at 365 nm (a) and at 254 nm (b); Figure S8: HPLC chromatogram of the isolated compound; Figure S9. Positive ESI–MS spectrum analysis of the isolated compound; Figure S10: The relationship between the inhibition zones against *Candida albicans* ATCC10231, corresponding to various antifungal metabolite concentrations; Figure S11: The relationship between the UV absorbance at λmax at 273 nm and various concentrations of antifungal metabolite in µg/mL.
