# Supplementary material for: Identification, Characterization, and Production Optimization of 6-Methoxy-1H-Indole-2-Carboxylic Acid Antifungal Metabolite Produced by Bacillus toyonensis Isolate OQ071612
Source: Microorganisms. 2023 Nov 22;11(12):2835. doi: 10.3390/microorganisms11122835 (PMC10745709; doi:10.3390/microorganisms11122835)
Supplement: Supplementary file 1 [file microorganisms-11-02835-s001.zip › microorganisms-2624440-supplementary.pdf]

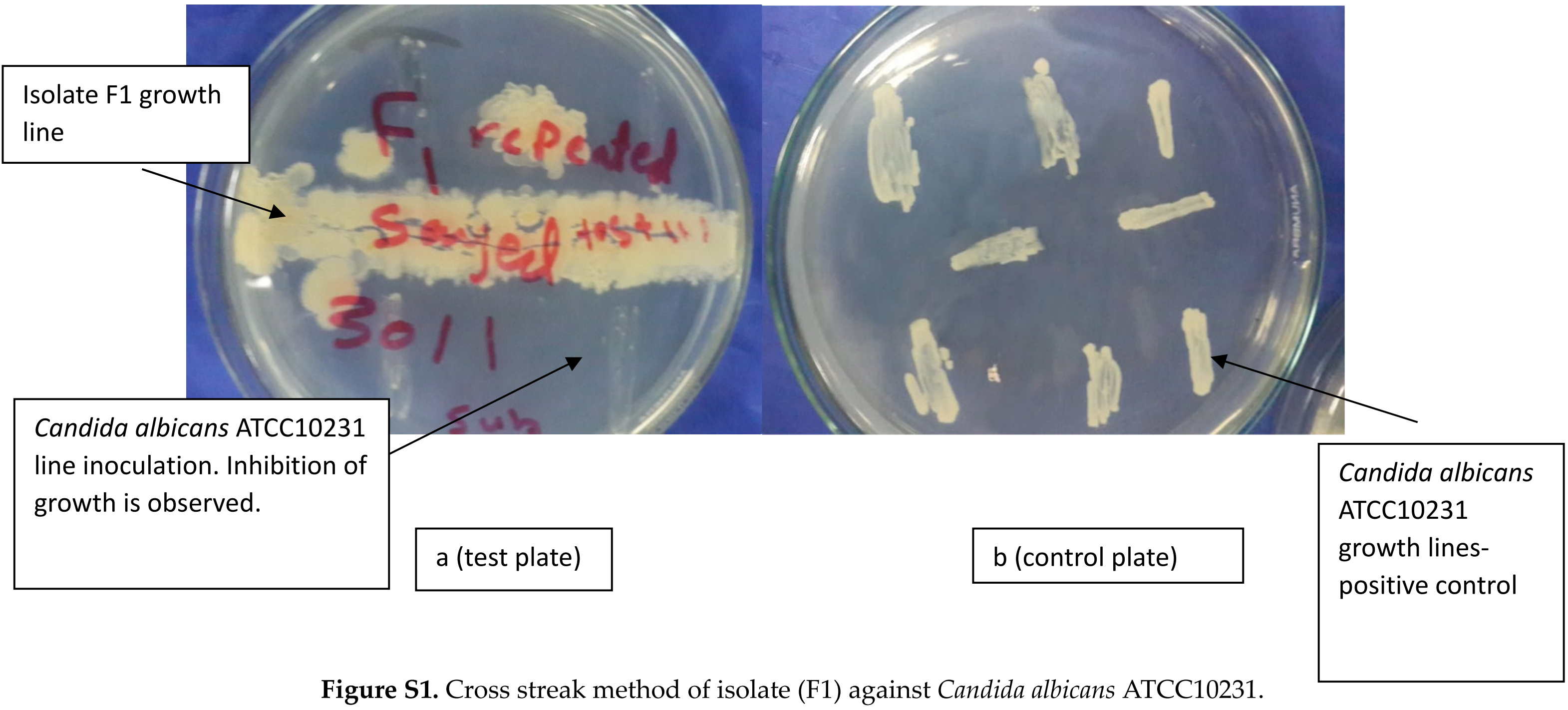

Figure S1. Cross streak method of isolate (F1) against *Candida albicans* ATCC10231.

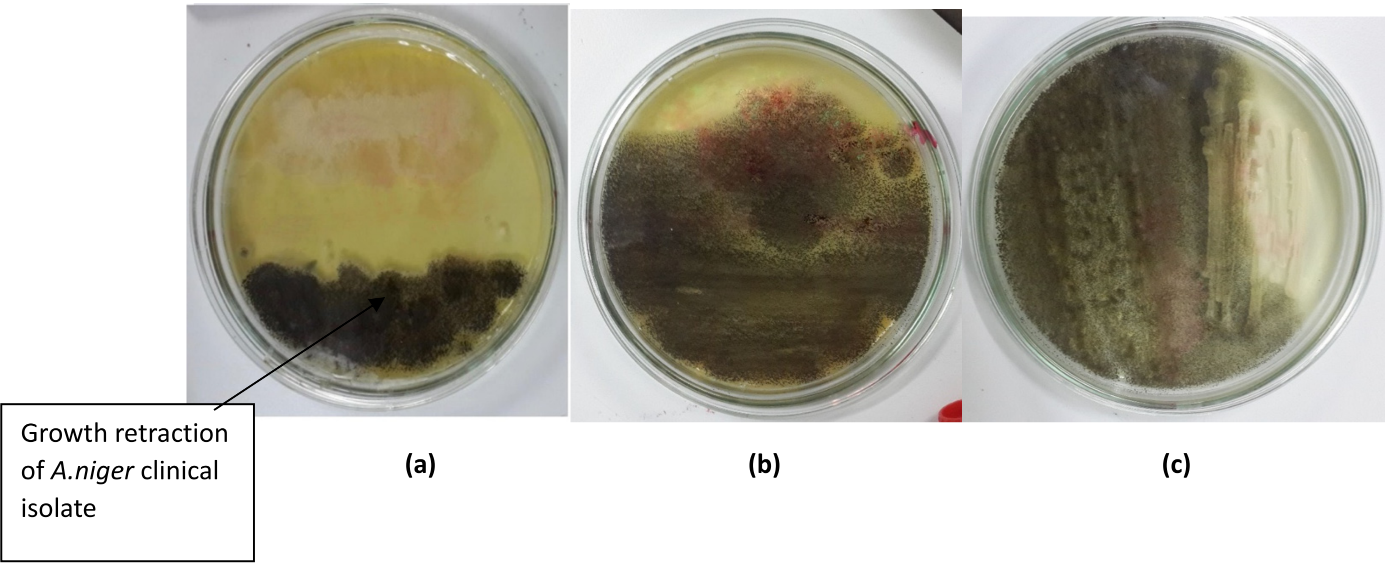

Figure S2. Dual culture technique showing inhibitory effect of isolate (F1) against *A. niger.* (a) shows clear zone between the edges of fungal mycelia and bacterial colonies. (b) the control, inoculated with *A. niger* without streaked bacteria (c) control showing the diffuse growth of *A.niger* in the presence of a non-productive bacteria.

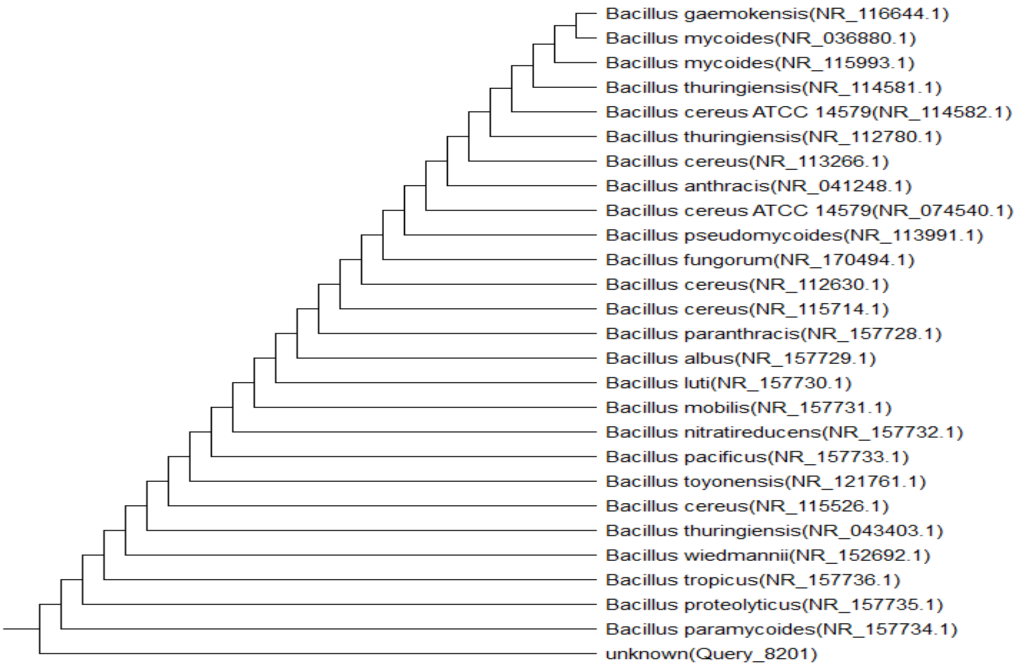

Figure S3. Molecular phylogenetic analysis of the query isolate *Bacillus toyonensis* OQ071612 strain (F1) using Maximum Likelihood method based on the Tamura-Nei model in MEGA X.

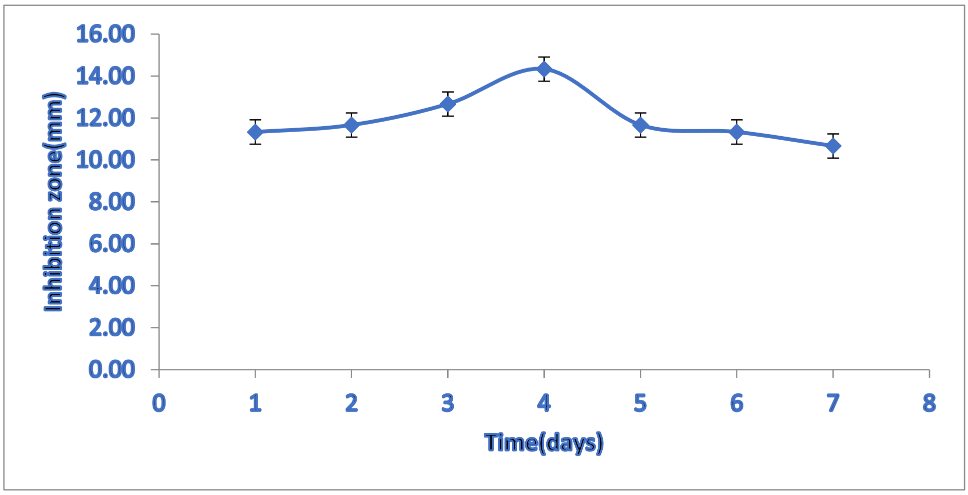

Figure S4 . Effect of incubation time on the yield of the antifungal metabolite produced by *Bacillus toyonensis* OQ071612 against *Candida albicans* ATCC10231.

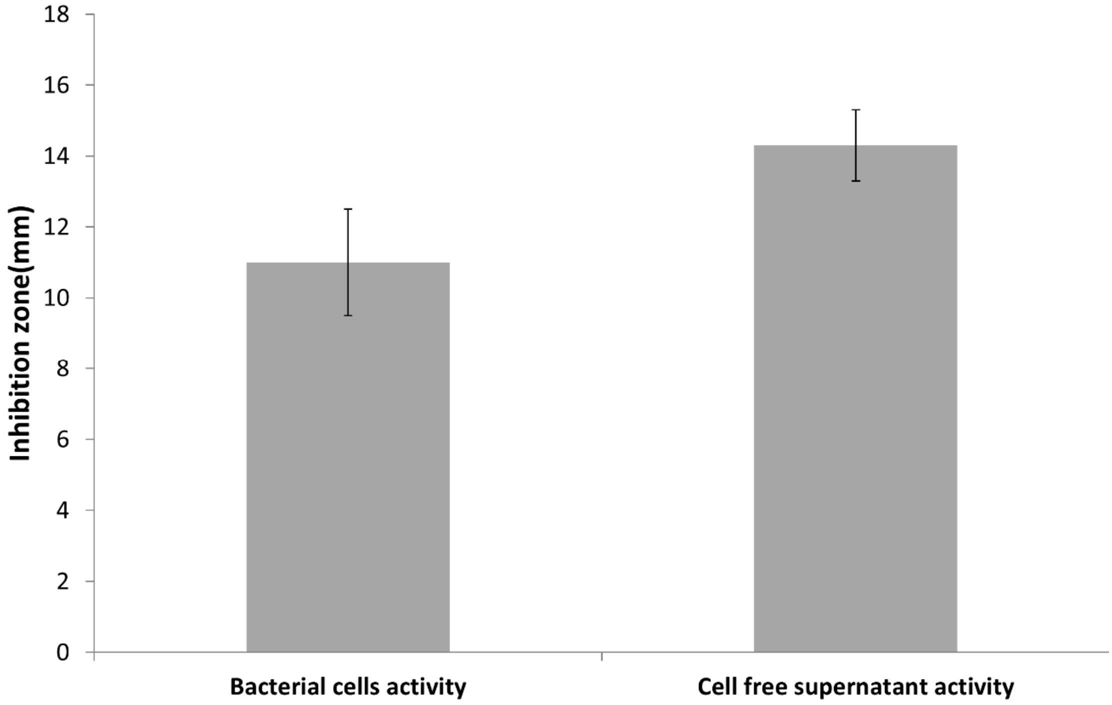

Figure S5. Average inhibition zones of the tested intracellular and extracellular antifungal metabolite(s) produced by *Bacillus toyonensis* OQ071612 against *Candida albicans* ATCC10231.

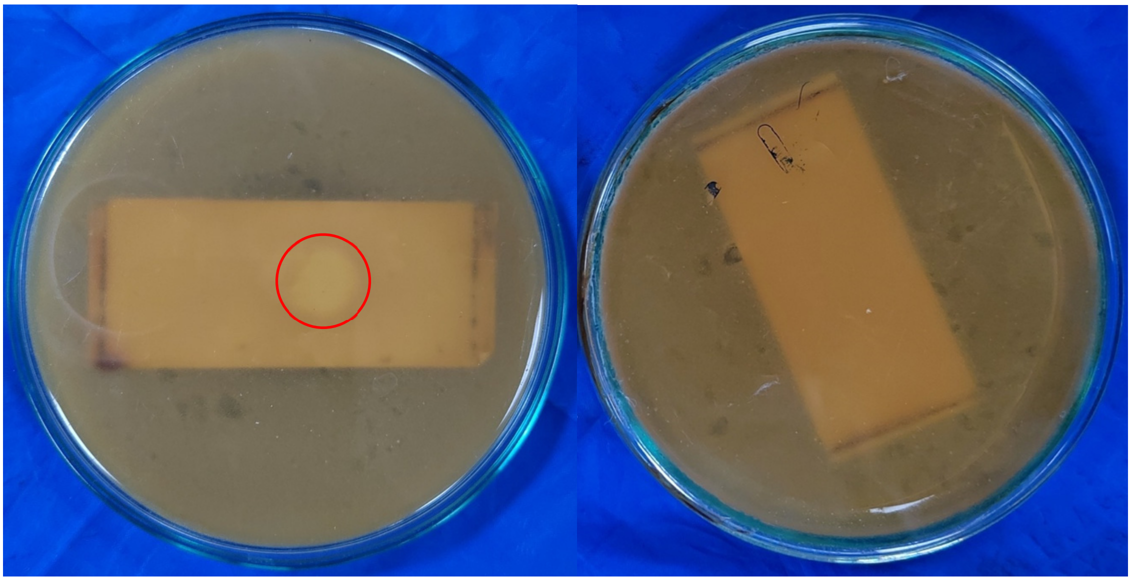

(a) **(b)**

Figure S6 The bioautography method was used to determine the antifungal activity of the pooled fractions obtained from column chromatography fractionation against *C. albicans*. Plate (a) highlights the zone of inhibition caused by the fractions of the *B. toyonensis* metabolite with the most potent antifungal activity. Plate (b) control plate.

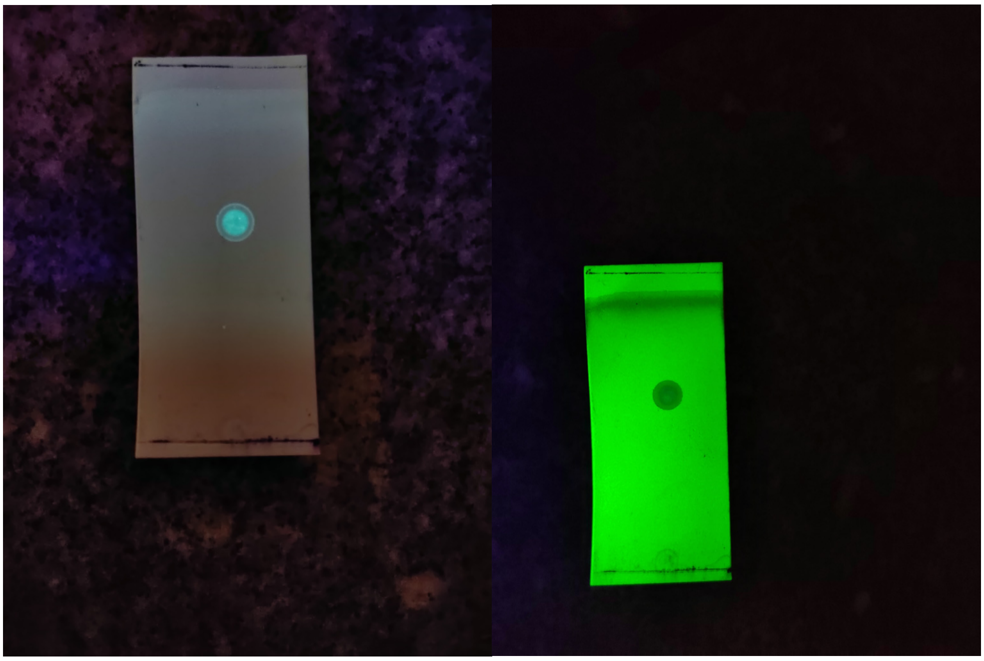

a b

Figure S7. Visualization of the most active pooled fractions of the antifungal metabolite of *B. toyonensis* isolate OQ071612 under UV lamp (UVitec®) at 365 nm (a) and at 254 nm (b).

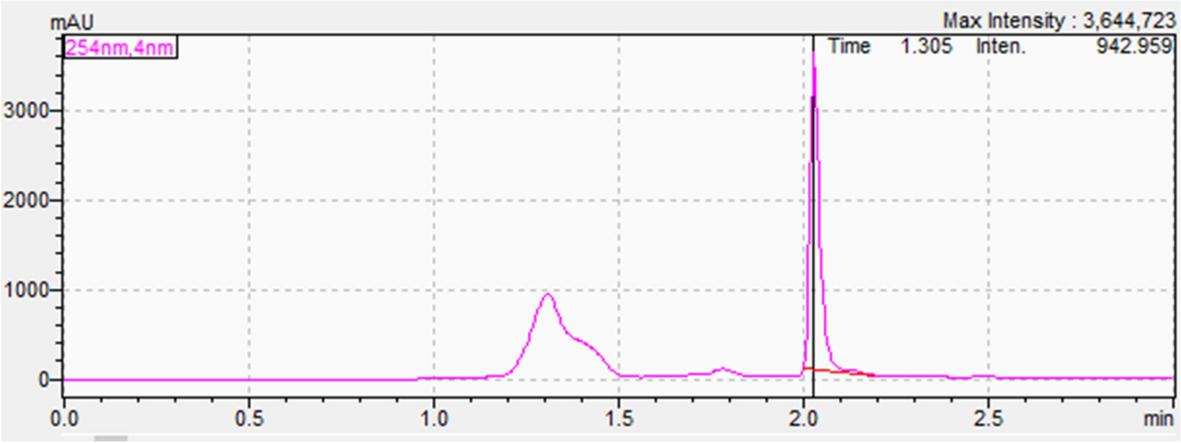

Figure S8. HPLC Chromatogram of the isolated compound.

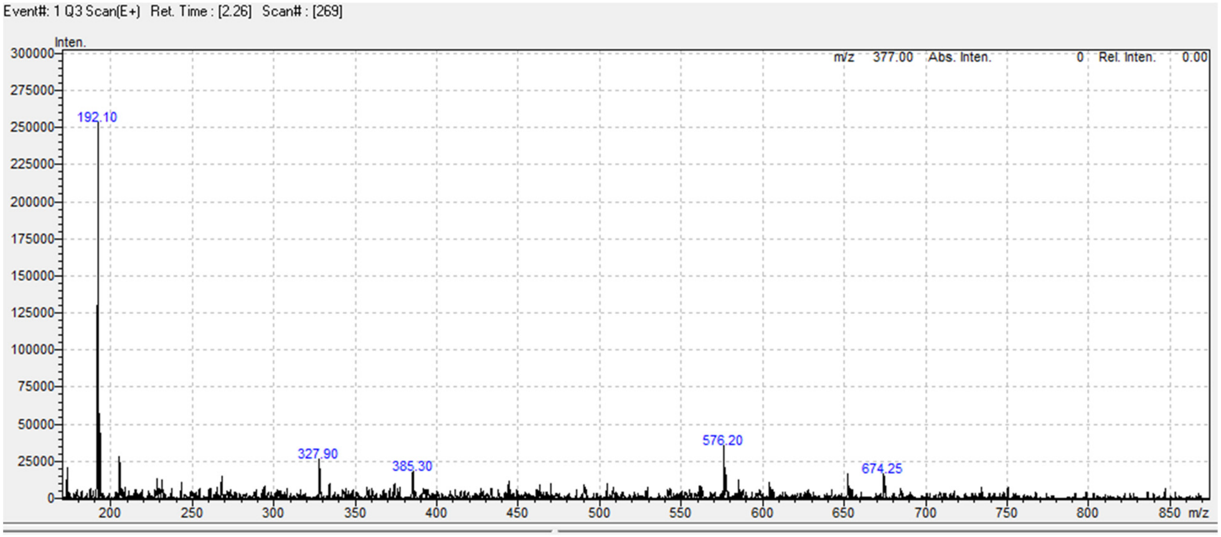

Figure S9. +ve ESI-MS Spectrum of the isolated compound.

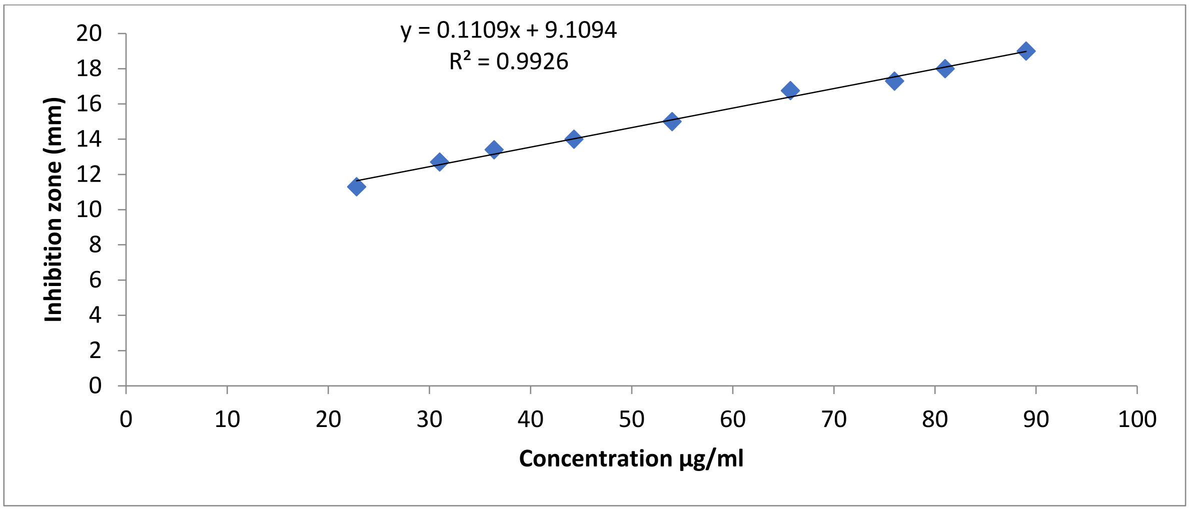

Figure S10. The relationship between the the inhibition zones against *Candida albicans* ATCC10231 corresponding to various antifungal metabolite concentrations.

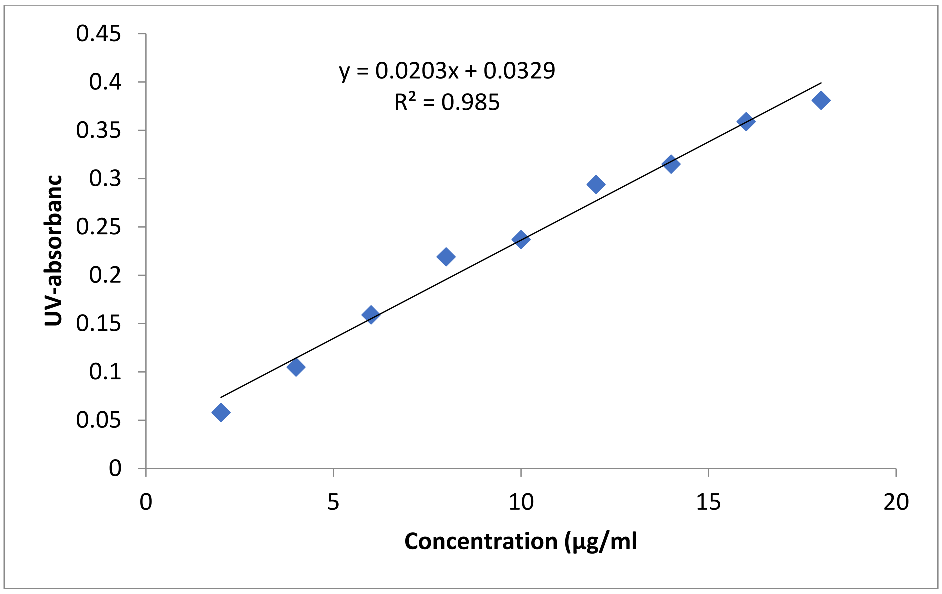

Figure S11. The relationship between the UV absorbance at λmax at 273 nm and various concentrations of antifungal metabolite in µg/mL.
